# Kaposi sarcoma-associated herpesvirus cooperates with Epstein-Barr virus to co-transform a small set of human B cells oncogenically

**DOI:** 10.1371/journal.ppat.1013281

**Published:** 2025-06-23

**Authors:** Mitchell Hayes, Wei Wang, Isabela Fraga de Andrade, Paul F. Lambert, Bill Sugden

**Affiliations:** 1 McArdle Laboratory for Cancer Research, University of Wisconsin–Madison, Madison, Wisconsin, United States of America; 2 Department of Microbiology, Genetics, & Immunology, Michigan State University, East Lansing, Michigan, United States of America; Wistar Institute, UNITED STATES OF AMERICA

## Abstract

KSHV has not been found to transform human B cells alone. We show now it infects both peripheral and tonsillar B cells inefficiently and cooperates with EBV to co-transform a similar, small fraction of cells from both sources. These cells yield immortalized progeny, one hallmark of transformation, and depend on both viruses for their continued growth. The cells secrete multiple cytokines that support their paracrine growth and express viral genes that mediate expression of these cytokines. The co-transformed cells grow preferentially as tumors in the peritoneal cavities of NSG mice outgrowing B cells transformed only by EBV. The levels of EBV genes expressed in co-transformed cells decrease during growth *in vitro* and more so during tumor growth *in vivo* while those of KSHV increase, mirroring that found In Primary Effusion Lymphoma (PEL) derived cell lines. The expression of cellular genes changes too, to reflect that of PEL biopsies. Both KSHV and EBV oncogenes are expressed in the co-transformed cells that regulate the gene signature of PELs, making these co-transformed cells a tractable model with which to understand this unique lymphoma associated with these two tumor viruses. Studies of this model will also illuminate the individual contributions of each virus to the many cancers they cause.

## Author summary

Primary Effusion Lymphoma (PEL) is an aggressive cancer of B cells that, by definition, is infected with Kaposi sarcoma-associated herpesvirus (KSHV). Remarkably, most PEL biopsies are also infected by a second herpesvirus, Epstein-Barr virus (EBV). Multiple groups have infected B cells with KSHV but not found them to be transformed. EBV, on the other hand, has been used to transform B cells for decades. We therefore posited that EBV and KSHV can cooperate to transform B cells isolated from peripheral blood and the tonsils. We describe the co-transformation and characterization of a small, discrete set of peripheral and tonsillar B cells with KSHV and EBV. The co-transformed cells can yield immortalized progeny that we show express and depend on transforming genes of each virus. We have found that they grow preferentially as tumors in the peritoneal cavities of NSG mice where the expression of both their viral and cellular genes is altered to mirror better that of Primary Effusion Lymphoma (PEL) biopsies. Multiple additional properties of co-transformed cells are shared with PEL cell lines making them informative models with which to understand PEL, the only cancer known to be caused by two tumor viruses.

## Introduction

Both EBV and KSHV cause B cell lymphomas, including Burkitt Lymphoma and Primary Effusion Lymphoma [1–3]. Early research with EBV demonstrated that it infects human B cells efficiently and transforms them [4,5], leading eventually to EBV-positive, immortalized cell lines in which EBV is maintained extrachromosomally [6]. These findings allowed characterization of the contributions of EBV to this cellular transformation and to the progression of infected cells to lymphomas. KSHV does not infect B cells efficiently, making it difficult to assess its role in transforming them. It can infect some tonsillar B cells that can be maintained with the addition of CD40 ligand and IL4 for limited times [7,8]. We found that we could infect a small subset of peripheral B cells with KSHV and that co-infection with EBV supported their continuous growth in the absence of added CD40 and IL4 [9]. We now have infected tonsillar B cells with KSHV and find that only when co-infected with KSHV and EBV can a small fraction of them grow as can co-infected peripheral B cells.

KSHV and EBV have not been found to co-infect B cells from other species, so the human co-infected peripheral and tonsillar B cells are now the only model for this cooperative transformation [9,10]. These co-infections therefore could exemplify the majority of Primary Effusion Lymphomas which also are B cells co-infected with KSHV and EBV [2,3]. We have examined these model cells to characterize their growth requirements *in vitro*, their tumorigenicity *in vivo*, and contributions each virus makes to these cells to assess their similarities to PELs. The co-infected cells can be cloned, yield co-transformed, immortalized progeny, and maintain both KSHV and EBV as plasmids indicating a dual viral requirement for the survival and/or proliferation of these cells [11,12]. Accordingly, targeting the RNAs encoding transforming genes of either virus with CRISPR/RfxCas13d in co-transformed cells inhibits their growth. The co-transformed cells secrete multiple cytokines, including IL-6 and IL-10, that foster their paracrine growth. Our RNA measurements show that both KSHV and EBV express genes that mediate the secretion of these cytokines [13–15]. In addition, both KSHV and EBV express their miRNAs in the co-infected cells extremely efficiently so that they represent 40% of the total miRNAs in co-infected cells. These miRNAs regulate cellular pathways critical for lymphomagenesis including inhibiting apoptosis [16–18].

Our new studies demonstrate that the co-transformed cells grow preferentially as tumors in the peritoneal cavities of NOD scid gamma (NSG) mice outgrowing cells transformed by EBV alone. Propagating them in this body cavity also alters their gene expression to mirror better that of PEL biopsies [19]. RNA-seq analysis shows that PEL cell lines usually express a small subset of EBV genes while the co-transformed cells derived in this study initially express more EBV genes *in vitro*. The levels of expression of some of these EBV genes decreased as the co-transformed cells proliferated to yield immortalized progeny. This decrease became more pronounced when the co-transformed cells grew as tumors in the peritoneal cavities of NSG mice. This change in viral gene expression *in vivo* arises from a decreased use of the Bam C/Bam W promoters and not from an alteration in the promoters used for latent EBV transcripts. Propagating the co-transformed cells *in vivo* also altered the expression of cellular genes including those encoding the cytokines, IL-6 and IL-10, better reflecting that of PELs [14,15]. Importantly, these analyses of co-transformed cells illustrate their similarities to PEL cells and support the hypothesis that early stages of PELs secrete cytokines that act as paracrine drivers of survival and/or growth. They also have uncovered a critical role for the microenvironment of the peritoneal cavity in affecting viral and cellular gene expression to mirror better that of PEL biopsies and potentially mimicking the later progression of PELs in their lymphomagenesis. B cells transformed cooperatively by KSHV and EBV therefore provide an informative model to investigate the progression of PELs *in vitro* and *in vivo*.

## Results

### KSHV and EBV co-transform both peripheral and tonsillar B cells to yield immortalized progeny

**Fig 1:**
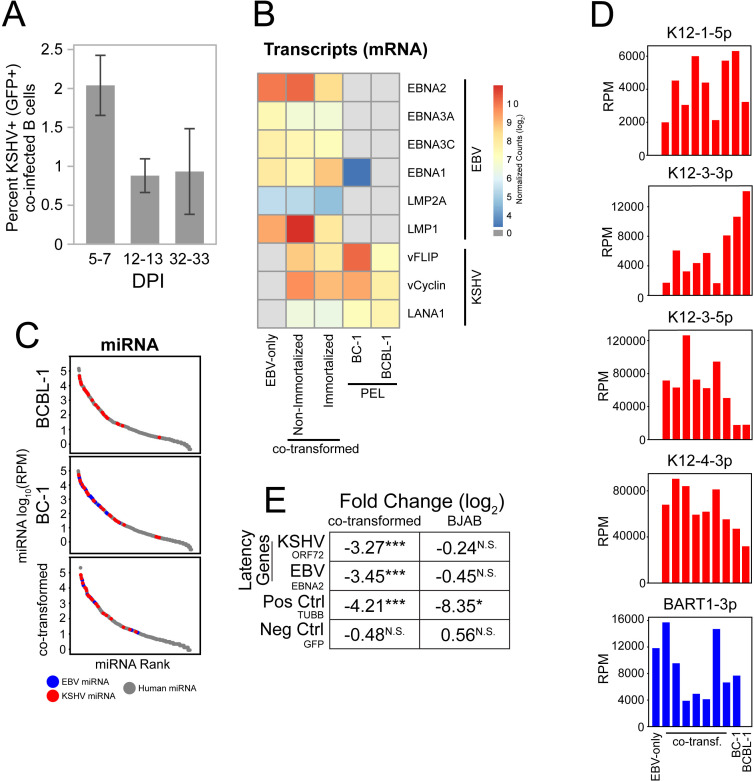
Co-infected cells express both KSHV and EBV genes *in vitro* necessary for their survival and growth. (A) The percent of tonsillar B cells infected with KSHV at three timepoints after exposure to EBV and KSHV was determined using flow cytometry. Shown are the means and standard deviations of 2 (5-7 DPI (days post infection), 12-13 DPI) and 4 (32-33 DPI) independent experiments. (B) The viral mRNAs expressed *in vitro* were measured by RNA-Seq and quantified as described previously [9]. (C,D) Small RNA-Seq of established PEL cell lines (BCBL-1, BC-1) and co-transformed cells was performed to assay the relative expression of host and viral miRNAs (KSHV: red, EBV: blue, and Human: gray) (C) and individual viral miRNAs (D) (y-axis: RPM (reads per million miRNAs) (E) CRISPR/RfxCas13d transcript targeting was used to test a requirement for ORF72 of KSHV and for EBNA2 of EBV for sustaining co-transformed cells over 10 doublings. KSHV-EBV- BJAB cells were used as a control cell line, TUBB as an essential human gene, and GFP as a non-essential target. (N.S.: not significant; *: p < 0.05; **: p < 0.01; ***: p < 0.001.).

Epstein-Barr virus and Kaposi sarcoma-associated herpesvirus can co-infect and co-transform a small fraction of peripheral B cells [9]. However, it is unknown whether these phenotypes of co-infection and co-transformation would extend to B cells isolated from other compartments. Several groups have infected tonsillar B cells with KSHV but have not reported transformation of these B cells (continued growth for more than 20 population doublings in the absence of supplemented human cytokines). We therefore extended our work with peripheral B cells to learn if tonsillar-derived B cells can be co-infected and also transformed by the combination of KSHV and EBV. We co-infected tonsillar B cells from four donors with cell-free EBV (B95-8) and KSHV (BAC16-GFP) at MOIs of 2–4 for each virus. Without EBV co-infection, only 0.1% of cells were infected by KSHV at 12 days and failed to proliferate. When co-infected with EBV, ~ 2% of exposed cells were infected with KSHV by seven days as measured by flow cytometry (Fig 1A) and co-infected cells continued to grow. These co-infection rates are similar to those for peripheral B cells. Importantly, no cells grew in the absence of infection with EBV. Both peripheral and tonsillar co-transformed cells were cloned with 50-fold increased efficiencies on fibroblast feeder layers. Both also expressed λ-light chain as measured by RNA-seq and flow cytometry as shown by Totonchy, et. al. [20] (S1 Fig). These findings provide one mechanistic insight into co-transformation of B cells by KSHV and EBV: While KSHV can infect both peripheral and tonsillar B cells *in vitro*, it cannot drive their proliferation in the absence of added cytokines, while co-infection with EBV can. Most PEL cell lines are dependent on EBV too [21,22]. Clones of co-transformed cells were propagated for more than 100 doublings (used hereafter as a threshold for immortalization), demonstrating for the first time that KSHV and EBV can cooperate to immortalize B cells *in vitro*.

B cells isolated from peripheral blood or tonsils, once co-infected, behave similarly and thus are likely derived from a similar subset of B cells. They can readily be expanded to 10^8^ cells, frozen viably, thawed and expanded again. We cloned one set of co-infected, peripheral B cells after two rounds of extended growth, followed each time by sorting with FACS for GFP + co-transformed cells, and isolated a co-transformed clone that grows effectively indefinitely (more than 100 population doublings). In general, though, co-transformed B cells cease to proliferate after extended growth. We engineered and used a retrovirus that expresses both hTERT and RFP to overcome this limitation. For example, in parallel experiments, one clone of co-transformed, peripheral B cells and one of tonsillar B cells were infected either with the retrovirus encoding hTERT or its parent that expresses only RFP, the four infected populations isolated by FACS, plated on human fibroblasts, and followed. By six to eight weeks after the sorting, all the cells infected with the RFP-only virus had died while those infected with the virus encoding hTERT continued to grow indefinitely. The indefinitely growing clones continued to maintain both KSHV and EBV genomes (S2 Fig). These experiments show that infection of co-transformed B cells with an hTERT virus can extend their growth efficiently and indefinitely, simplifying their long-term study. Clones of co-transformed cells that were spontaneously immortalized or by infection with an hTERT-expressing retrovirus maintain both viral genomes as measured by FISH (S2 Fig) demonstrating that both KSHV and EBV provide these model cells transforming functions beyond immortalization.

### Co-transformed cells express and depend on EBV and KSHV transforming genes *in vitro*

We measured gene expression in PEL cell lines and co-transformed cells by RNA-seq. The profile of EBV gene expression of co-transformed cells was found to be closest to latency III in which EBV’s transforming genes are all expressed [23] (Fig 1B and S1 Table). Co-transformed cells also expressed the latency genes of KSHV. These analyses demonstrate that both KSHV and EBV express transforming genes in these cells known to support cell survival and growth [1,24]. Early passage, co-transformed cells expressed their EBV genes at higher levels than do the cells that grew to be immortalized (Fig 1B and S1 Table). In addition, the viral miRNAs were abundantly expressed with approximately 10% of all miRNAs expressed by EBV and 30% by KSHV (Fig 1C). These levels are similar to those detected in tumor biopsies (1–24% reads in two BLs were EBV miRNAs [25]; 9–44% of reads in two PEL cell lines were encoded by KSHV [26]). They indicate that co-infected cells express viral miRNAs *in vitro* at levels found in PEL cell lines. In particular, viral miRNAs that have previously been reported to promote the survival of both KSHV and EBV transformed cells by inhibiting apoptosis (including miR-K12-1, 3, and 4–3 encoded by KSHV and BART 1-3p encoded by EBV) [16–18] were expressed at similar levels in co-transformed cells and PEL cell lines (Fig 1D). The co-expression of the transforming genes and miRNAs of KSHV and EBV in co-transformed cells illustrates their dual roles in maintaining survival and/or growth. This dual role was confirmed by targeting latency transcripts including the ORF72 transcript of KSHV encoding vCyclin and the transcript of EBNA2 of EBV in a co-transformed cell clone with CRISPR/RfxCas13d and performing a competitive growth assay. The co-transformed cells in which transcripts encoding transforming genes of both viruses were targeted exhibited an 8–11 fold growth/survival defect relative to control guides (Fig 1E) demonstrating their dual dependence on KSHV and EBV. EBNA2 is essential for the transformation of B cells by EBV and vCyclin promotes survival of PEL cell lines [27–29]. These analyses of viral RNAs and their required roles illustrate the enigma central to most PELs and shared with the co-transformed cells that model them: while KSHV and EBV can each independently cause tumors, both are required in these transformed B cells.

### Co-transformed cells can support their growth through paracrine signaling

**Fig 2:**
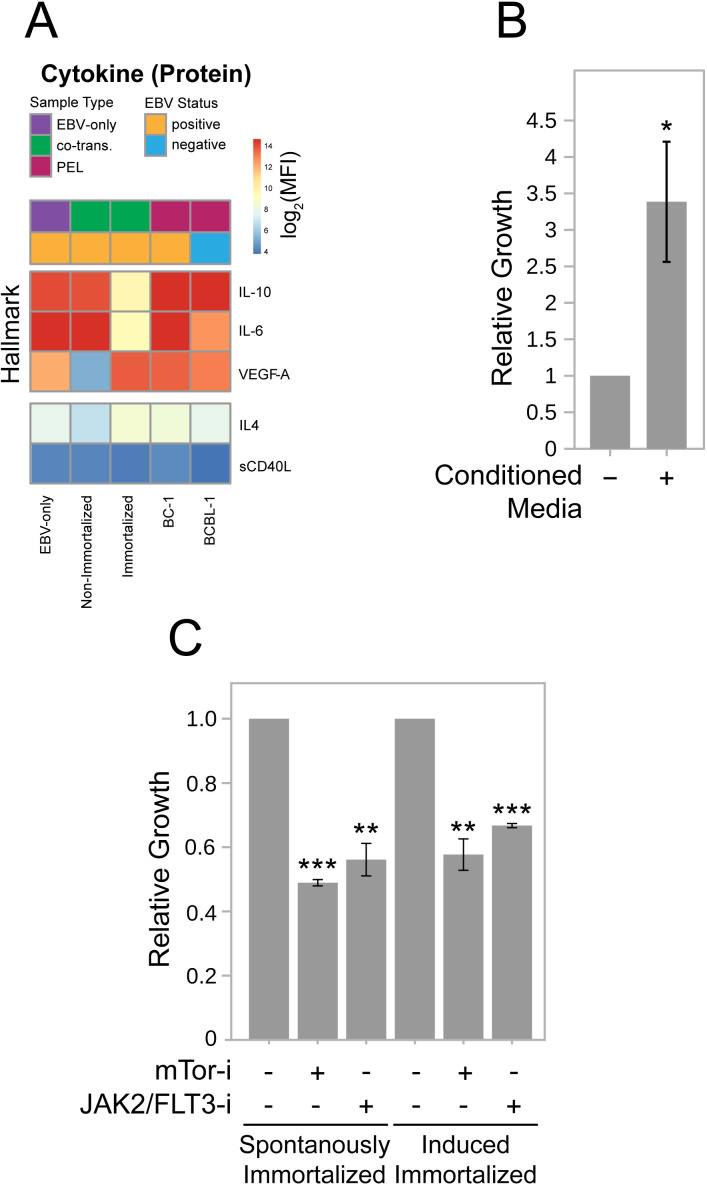
Co-transformed cells secrete cytokines that can support their growth through JAK/STAT signaling. (A) Cytokines known to support the growth of B cells were measured in conditioned media from cells infected only with EBV, from non-immortalized and immortalized co-transformed cells, and from established PEL cell lines (KSHV^+^EBV^+^ or KSHV^+^EBV^-^). (B) Co-transformed cells were grown at a density of 5X10^4^ cells/ml in basal medium or in 50% conditioned medium. (*: p < 0.05) (C) Co-transformed cells were grown in complete medium or complete medium supplemented with rapamycin (100 nM) or pacritinib (100 nM) for 48 hours. (**: p < 0.01; ***: p < 0.001).

PEL cell lines secrete cytokines and respond to them by paracrine growth [14,15]. We asked if the co-transformed cells model this feature of PEL cells too, by assessing their growth requirements in minimal media conditioned by two clones of co-transformed cells. The compositions of the different conditioned media, along with media conditioned by the established PEL cell lines BC-1 (KSHV+EBV+) and BCBL-1 (KSHV+EBV-) and by cells transformed by EBV alone were determined with a multiplex Luminex assay (Fig 2A and S2 Table). The compositions of the media conditioned by either PEL cell lines or co-transformed cells were comparable but varied in the relative concentrations of some constituents. Our RNA assays failed to detect RNAs encoding IL4 or CD40L in the co-transformed cells while the Luminex assays of their conditioned medium measured IL4 to be ~ 2 pg/ml, that is, less than 0.1% of that used to sustain KSHV-infected B cells in culture [7,8] and sCD40L was below the lower limit of detection. These assays detected IL-6 at levels of 0.01-1.5 ng/ml and IL-10 at levels of 0.1-2 ng/ml in the conditioned media both of which have been found to support the growth of PEL cell lines [14,15]. The co-transformed clones grew in their conditioned media (Fig 2B), demonstrating they share the property of paracrine growth with PEL cell lines, that this growth is independent of IL4 and CD40L, but dependent on factors they secrete.

PEL cell lines can be induced to grow by the addition of IL-6 and IL-10 [14,15]. This response requires signaling through mTOR which can be inhibited by rapamycin [30] and MLN0128 [31]. Signaling by IL-6 and IL-10 is mediated through JAK/STAT pathways which are inhibited in PEL cell lines by the small molecule, pacritinib [32]. Treatment of clones of co-transformed cells with both rapamycin and pacritinib inhibited their growth in complete medium (Fig 2C) showing that the co-transformed cells model the signaling requirements for growth of the PEL cell lines. That both B cells cooperatively transformed by KSHV and EBV and PEL cell lines share these requirements indicates that paracrine signaling is likely to contribute to the early progression of PELs.

### Co-transformed lymphocytes are tumorigenic in the peritoneum *in vivo*

**Fig 3:**
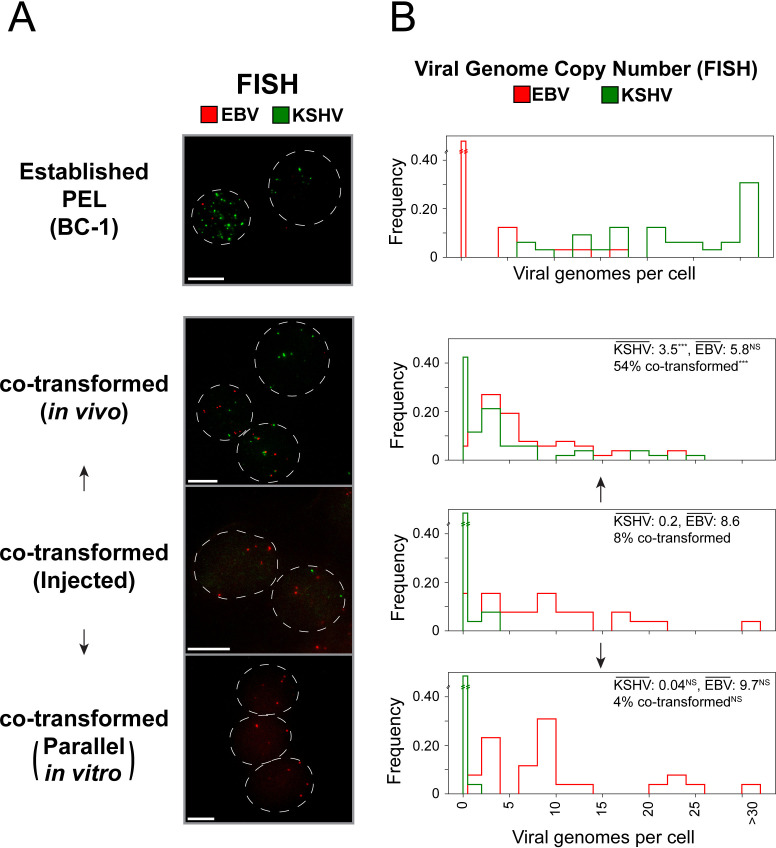
Co-transformed cells grow preferentially as tumors in peritoneal cavities. A population of co-transformed B-cells and B-cells infected with EBV alone were either injected into the peritoneal cavities of NSG mice or passaged in parallel *in vitro* to assess their relative growth. After 23 days, the parallel cultures were harvested and the KSHV and EBV copy number measured by FISH. (A) Fluorescence micrographs of the PEL cell line BC-1 (KSHV^+^EBV^+^) grown *in vitro* and the B cell populations injected *in vivo* or passaged in parallel *in vitro*. The KSHV and EBV genomes were detected simultaneously by FISH (KSHV probe (green, FITC) and EBV probe (red, Cy3) with nuclei indicated by dashed grey outlines (Zeiss Plan Apo 63X/1.4, Scale bars: 10 microns). (B) Quantification of viral genome copy number from FISH performed in (A). Mean viral genome copy number and proportion of cells co-transformed is shown for each sample. Differences in viral genome copy number and proportion of cells co-transformed between each sample and the injected sample were determined using Wilcoxon Rank-Sum Test and Barnard’s exact test, respectively (*** p < 0.001, NS: not significantly different).

A characteristic clinical feature of PELs is their preferential growth in body cavities [33]. We asked if co-transformed cells are tumorigenic and share any preferred growth in body cavities. We found that both PEL cell lines and co-transformed cells grew as tumors in the peritoneal cavities of NSG mice. For example, 6 of 6 xenografts of established PEL cell lines and 7 of 7 xenografts of spontaneously immortalized co-transformed cells grew as tumors in these mice. It is known that EBV-infected B cells grow well as xenografts in the peritoneum of immunodeficient mice [34]. To test for growth in the peritoneal cavity of co-transformed cells relative to those infected with EBV alone, we injected a population of co-transformed and EBV-only infected B cells derived from exposure of B cells to EBV and KSHV. At the time of injection, the EBV-only infected B cells greatly outnumbered the co-transformed B cells (approximately 8% of the cells were co-transformed). This population was injected into mice to form ascites tumors in the peritoneum or grown *in vitro* (Fig 3A and 3B). By 23 days the co-transformed cells injected *in vivo* had outgrown the EBV-only B cells and represented 54% of the population (p < 0.001, Barnard’s exact test). For the population grown *in vitro*, the co-transformed cells were outgrown by those transformed by EBV alone, so that the co-transformed cells constituted less than 5% of the population. Thus, growth *in vivo* increased the fraction of co-transformed cells approximately 10-fold relative to those grown in parallel *in vitro*. Growth as tumors *in vivo* also increased the average number of KSHV genomes in the co-transformed cells but maintained the average number of EBV genomes (Fig 3A and 3B). Most of the tumor cells varied in their numbers of each viral genome illustrating their maintenance of these genomes extrachromosomally. The microenvironment of the peritoneal cavity strongly favors the survival/growth of co-transformed cells giving us an important mechanistic insight into co-transformation and the likely progression of PELs: the body cavity’s microenvironment favors their survival and growth.

### *In vivo* viral gene expression supports tumorigenesis

We analyzed PEL cells as xenografts in NSG mice to provide a baseline for the analysis of co-transformed cells. Two such cell lines, BC-1 and BCBL-1, were injected into the peritoneal cavities of NSG mice to assay their tumorigenicities and gene expression when grown in cell culture and as tumors. Both BC-1 (KSHV^+^EBV^+^) and BCBL-1 (KSHV^+^EBV^-^) grew rapidly and were harvested from the peritoneal cavity. The differences in gene expression between *in vitro* samples and harvested tumors were compared to the differences previously found by Klein et al. [19] that distinguish PEL biopsies from Burkitt Lymphomas (BLs) and Diffuse Large B Cell Lymphomas (DLBCLs). The gene set containing human genes whose expression was higher in PEL than the other B cell malignancies (“PEL Up”) was enriched by GSEA in the two PEL cell lines when grown *in vivo* compared to being grown *in vitro* (S3 Fig). These results indicate that the microenvironment of the peritoneal cavity supports *bona fide* PEL cell lines evolving their gene expression to represent more closely the tumors from which they were derived.

**Fig 4:**
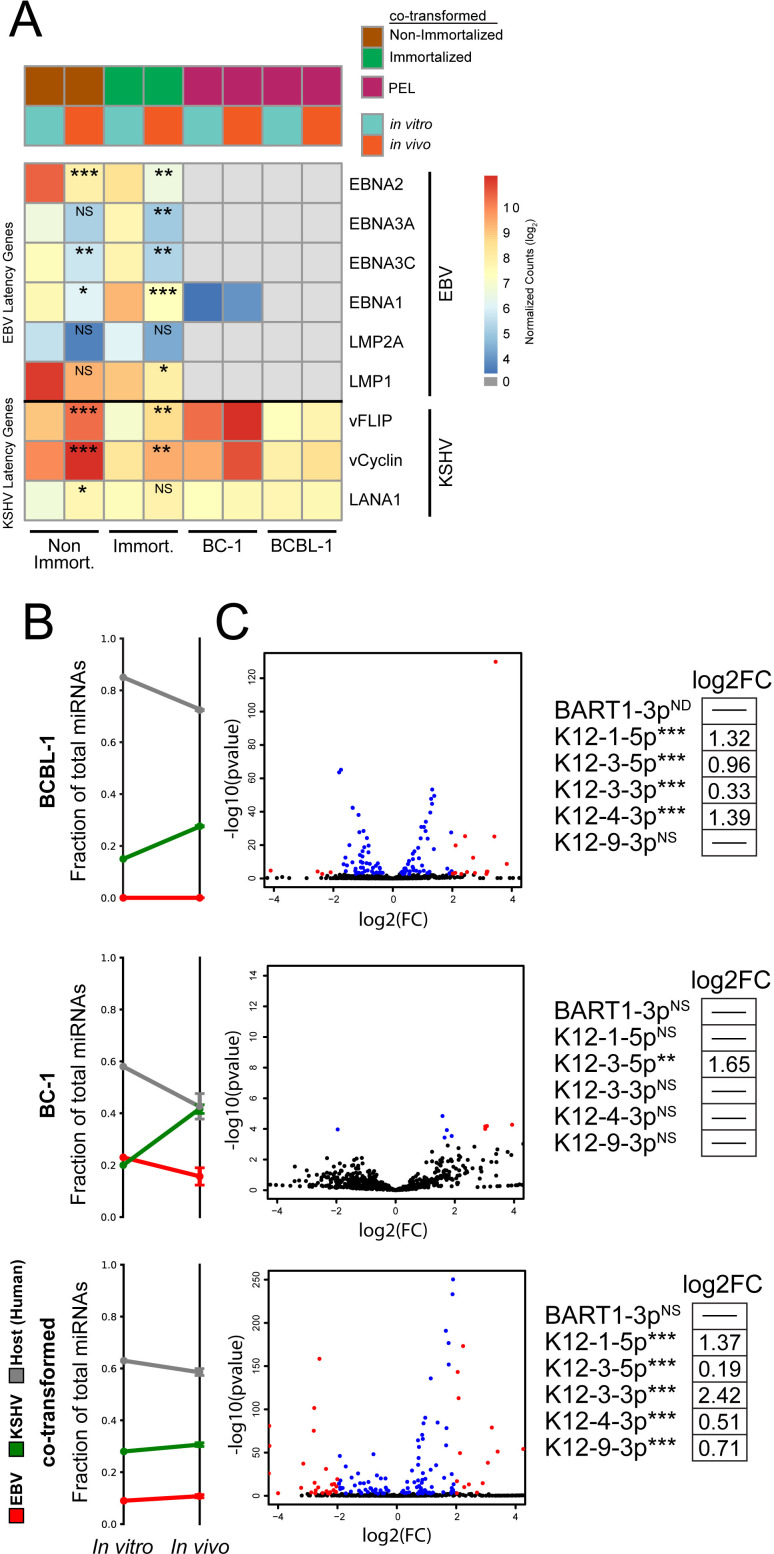
The growth of co-transformed cells in the peritoneal cavity alters viral gene expression to mirror that of PEL cell lines. The differential viral expression of co-transformed cells engrafted into the peritoneal cavities of NSG mice was measured by mRNA-seq in harvested tumors and compared to that of the co-transformed cells *in vitro*. (A) Shown are the normalized expressions of EBV and KSHV latency gene transcripts from *in vitro* or *in vivo* passage of non-immortalized or immortalized co-transformed cells or the established PEL cell lines BC-1 (KSHV^+^EBV^+^) and BCBL-1 (KSHV^+^EBV^-^). Statistical significance of differential gene expression (*in vivo vs in vitro*) was determined by DESeq2 (* p < 0.05, ** p < 0.01, *** p < 0.001, NS: not significantly different). Viral miRNA expression was quantified with miRge from Small RNA-Seq analysis. (B) The fraction of total miRNAs expressed from EBV or KSHV genomes *in vitro* and *in vivo* are shown. (C) Differentially expressed miRNAs (blue: |log_2_FC| < 2, p-value<0.001; red: |log_2_FC|>=2, p-value<0.001) when established PEL cell lines or co-transformed cells were passaged in vivo. Individual viral miRNAs known from previous studies to target cellular apoptotic genes are shown. Fold changes are provided for viral miRNAs with a statistically significant expression difference determined by miRge (** p < 0.01, *** p < 0.001, NS: not significantly different).

We therefore could ask: Do co-transformed cells also alter their gene expression characteristically when grown as tumors in the peritoneum? We answered this question with two different approaches to assess any essential role for KSHV’s lytic genes in this model of tumorigenesis. We tested both co-transformed cells having intact KSHV and co-transformed cells that have a spontaneous deletion of much of KSHV’s lytic genes (the deletion spans from ORF9 to ORF44 and removes both the minor and major capsid proteins [35]) as xenografts. Both sets of co-transformed cells grew as tumors in the peritoneal cavities of the NSG mice indicating that the expression of the deleted set of KSHV’s lytic genes is not required for tumor formation. Total RNA was isolated and gene expression determined by RNA-Seq from the co-transformed cells grown *in vitro* and from the tumors grown in the peritoneal cavities of NSG mice. PEL cell lines *in vitro* usually express few EBV genes and multiple KSHV genes [36]. They expressed similar patterns when grown as tumors (Fig 4A), however, the co-infected cells maintained or increased their expression of KSHV genes when growing as tumors. The co-transformed cells that had wild-type KSHV detectably expressed genes associated with the lytic cycle including vCCL2/vMIP II, vGPCR, vIL6, and vIRF2 (S1 Table). The co-transformed cells with a deletion in the late region of KSHV did not express these genes detectably, as would be expected, but also formed tumors in the peritoneal cavities. This tumorigenesis indicates that expression of these lytic genes is not essential for tumor formation and provides another mechanistic insight from this model of PELs.

The co-transformed cells decreased their levels of expression of EBV protein-encoding genes as they grew as tumors *in vivo* (Fig 4A and S1 Table). The expression of six of EBV’s transforming genes is shown and of these three significantly decreased both in co-transformed cells when growing as tumors and in immortalized clones growing as tumors: (EBNA1, EBNA2, and EBNA3C). The expression of two KSHV transforming genes (ORF71 and ORF72) increased significantly under the same conditions (Fig 4A and S1 Table). This change is surprising: it was thought that cells transformed by EBV *in vitro* express viral genes characteristic of latency III because of the absence of immune selection found *in vivo*; NSG mice would not provide such immune selection. This decrease of multiple latency III transcripts could be the result of decreased transcription from Cp/Wp, the initiation of transcription from Qp, or a combination of these mechanisms. To address which of these mechanisms underlies this change in gene expression we focused on the transcripts encoding EBNA1, which is expressed in latency I, II, and III (S4AFig). The EBNA1 ORF, in the K exon, is encoded by transcripts initiated at Cp, Wp, and Qp depending on the latency status of the genome. RT-PCR with exon-specific primers (shown in S4A Fig) was performed to quantify the relative abundance of Cp/Wp and Qp initiated transcripts containing the K exon. These measurements demonstrated that *in vivo*, these transcripts originate either from the Bam C or Bam W promoters [37]. These promoters were active in cells grown *in vitro* and in the peritoneal tumor cells, but the level of transcription from them decreased in the cells grown *in vivo* relative to those grown *in vitro* (S4 Fig).

Contrary to the changes observed in viral protein-coding transcripts, the levels of expression of both EBV and KSHV miRNAs did not change dramatically when co-transformed cells grew as tumors, remaining high and accounting for 10% and 30% of the total miRNAs in the cells respectively (Fig 4B and 4C). The miRNAs that have previously been shown to inhibit apoptosis (including miR-K12-1, 3, and 4–3 encoded by KSHV and BART 1-3p encoded by EBV [16–18]) either were maintained or increased in their expression in co-transformed cells grown *in vivo* (Fig 4C). That the microenvironment of a body cavity alters the viral gene expression of both EBV and KSHV in co-transformed cells to be closer to that of PEL biopsies explains its fundamental contribution to the development of this lymphoma.

### In vivo cellular gene expression supports tumorigenesis

**Fig 5:**
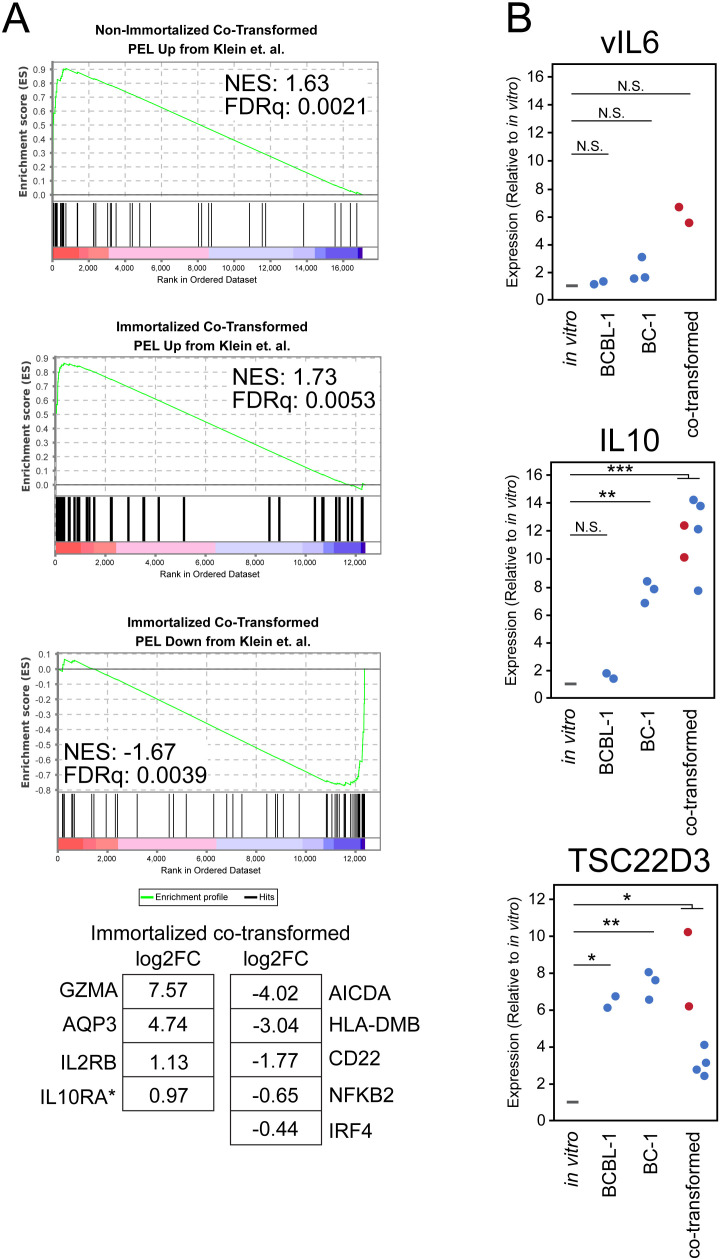
The growth of co-transformed cells in the peritoneal cavity alters their cellular gene expression to mirror that of PEL biopsies. The differential expression of cellular genes in co-transformed cells engrafted into the peritoneal cavities of NSG mice was determined through RNA-seq of harvested tumors and in co-transformed cells grown *in vitro.* Non-immortalized co-transformed cells were grown for approximately 40-50 doublings after co-infection; immortalized co-transformed cells were grown for more than 100 doublings after co-infection. The measured differential expression was compared to that identified by Klein et al. [19] to identify similarities with biopsies of PELs that distinguish them from other B cell lymphomas. (A) The results of Gene Set Enrichment Analysis (GSEA). Differential expression of selected gene set members are shown (log_2_FC determined by DESeq2 of differential expression, *in vivo* vs *in vitro*, in immortalized co-transformed cells). (B) The expression of transcripts of vIL6, IL10, and the IL10 responsive gene TSC22D3 (GILZ) in the established PEL cell lines BCBL-1 and BC-1 or co-transformed cells (non-immortalized (red) or immortalized (blue)) *in vivo* relative to expression *in vitro* (* p < 0.05, ** p < 0.01, *** p < 0.001, NS: not significant).

The differences in gene expression between cells grown *in vitro* and as tumors in the peritoneal cavity were compared to the differences previously found that distinguish PEL biopsies from BLs and DLBCLs [19]. Both increases and decreases in gene expression of co-transformed cells grown *in vivo* versus *in vitro* reflected those found to be characteristic of PEL biopsies as measured by GSEA (Fig 5A). The microenvironment of the peritoneal cavity therefore alters cellular gene expression of co-transformed cells to conform to that of PEL biopsies [19]. Among the genes whose increased expression in co-transformed cells is characteristic of PEL biopsies are *IL10*, its receptor, *IL10Rα, IL6,* granzyme A*, GZMA*, and aquaporin*, AQP3* (Fig 5A). *IL10*, its receptor, and *IL6* all can promote growth. *GZMA*, granzyme A, can be expressed particularly highly in lymphoma cell lines and can elicit pyroptosis in neighboring cells [38]. The KSHV encoded vIL6 was not significantly increased on growing the co-transformed cells in the peritoneal cavity (Fig 5B). Among the genes that decreased characteristically were *HLA-DMB* and *CD22* (Fig 5A). *HLA-DMB* is involved in loading peptides onto MHC class II molecules and *CD22* can inhibit BCR signaling. These decreases in gene expression are predicted to limit immune recognition of both co-transformed cells and *bona fide* PELs. In addition, the *AICDA* gene which is required for the rearrangement of k- to λ-light chain was also strikingly decreased in the co-infected cells when grown *in vivo* (Fig 5A).

The observed changes in gene expression resulting from growing the co-transformed cells *in vivo* were confirmed. Co-transformed cells that had been grown as tumors in the peritoneal cavities were harvested, propagated *in vitro*, and then re-injected into the peritoneal cavities of NSG mice. RNA was isolated from the co-transformed cells grown *in vitro* and once *in vivo* and from the tumors following the second engraftment. The differences in gene expression between these two conditions were compared to the differences reported between PEL biopsies and both BLs and DLBCLs [19]. The increases in gene expression for co-transformed cells grown twice sequentially as tumors were changed similarly to those found following a single passage in NSG mice as measured by GSEA (S5 Fig).

The changes of viral gene expression in immortalized tumors included a decrease in the level of LMP1. Expressing it conditionally has revealed that LMP1 regulates both survival and growth of EBV-infected B cells [39]. The decreased levels of LMP1 in the immortalized co-transformed cells growing as tumors is reflected functionally in significant decreases in expression of genes it regulates including *NFKB2* and *IRF4* (Fig 5A) [40]. This decrease is likely compensated by increased expression of *IL6* and *IL10*. Both have been found to be autocrine growth factors for PEL cell lines *in vitro* [14,15]. Their expression was increased when both PEL cell lines and co-transformed cells were grown as tumors in the peritoneal cavity (Fig 5B). PEL biopsies were initially characterized by their efficient expression of IL6 and *IL10* when compared to that of BLs and DLBCLs [19]. We also measured the levels of genes regulated by *IL10. IL10* induces expression of *IL2RB* in B cells. Its level increased in co-infected cells when grown as tumors (Fig 5A) as it also does in PEL biopsies relative to those of BLs and DLBCLs [19]. A second gene regulated by *IL10* that is likely important for the survival of infected B cells is *TSC22D3/GILZ* [41]. *TSC22D3/GILZ* is necessary for the survival of B cells in mice; importantly its mRNA increased 4–5-fold in co-transformed cells growing as tumors (Fig 5B). These measurements show that the microenvironment of the peritoneal cavity supports the growth of co-infected cells as tumors, eliciting increased expression of cellular genes including the autocrine growth factor, *IL10*, which can function to support both their survival and growth (S1 Table).

## Discussion

Both KSHV and EBV cause cancers by themselves. How they together co-transform a cell is thus both perplexing and intriguing. We have addressed this puzzle by co-infecting primary B cells with KSHV and EBV and examining them *in vitro* and *in vivo* to dissect their cooperative, oncogenic transformation. These analyses of cells co-transformed by KSHV and EBV illustrate their being PEL-like cells (PEL-LCs); that is, they share many properties with PEL tumor cell lines. 1–2% of peripheral [9] and tonsillar B cells can be co-infected, yield progeny that require both viruses to survive and/or proliferate, become immortalized, grow as tumors in the peritoneal cavity of NSG mice (Fig 3), and warrant being termed PEL-LCs.

Both KSHV and EBV genomes in PEL cell lines and in PEL-LCs are maintained as extra-chromosomal plasmids (S2 Fig). These viral genomes are only maintained as plasmids in proliferating cells if they contribute one or more selective advantages to the cells [11,12], illustrating their both being required for the survival and/or growth of the PEL-LCs. CRISPR/RfxCas13d targeting the viral latency transcripts, ORF72 (vCyclin) and EBNA2, substantiated this dual requirement (Fig 1E). Due to the complex and overlapping structure of the latency locus of KSHV [42], the guide RNAs directed against ORF72 (vCyclin), may also degrade transcripts encoding other latency products (including LANA1, vFLIP, and the miRNAs). Therefore, the CRISPR/RfxCas13d experiment demonstrates a dependence on KSHV but the required genes are yet to be defined. Clearly though, transforming genes of both KSHV and EBV are required to support the survival and/or growth of PEL-LCs.

The analyses of PEL-LCs have identified multiple functions KSHV and EBV provide them. First, early following co-infection, EBV supports the growth of PEL-LCs without a need for exogenously added cytokines. Second, the co-infected cells prior to immortalization express KSHV and EBV genes to inhibit apoptosis (Fig 1A), including v-FLIP [43,44], and viral miRNAs (Fig 1B and 1C) [16–18] illustrating that both viruses contribute to cell survival. Third PEL-LCs secrete cytokines that promote their paracrine growth (Fig 2A and 2B). These cytokines are regulated by both KSHV and EBV [13–15], providing advantages to PEL-LCs cooperatively. The PEL-LCs cells are inefficiently spontaneously immortalized or can be efficiently immortalized following infection with a retrovirus encoding hTERT. Both spontaneously immortalized and induced (ectopic hTert) immortalized co-transformed cells subsequently maintain KSHV and EBV yielding a fourth insight (S2 Fig). Both viruses contribute transforming functions to PEL-LCs beyond immortalization. Fifth, growing PEL-LCs as tumors in the peritoneal cavities of NSG mice alters the expression of both cellular and viral genes better mirroring their expression in PEL biopsies (Figs 1 and 4), but does not require KSHV’s lytic genes. The microenvironment of this body cavity supports an increased copy number of KSHV genomes (Fig 3B) and decreased levels of expressed EBV genes (Fig 4A). It specifically supports increased levels of cellular mRNAs, including those of *IL-10* and *TSC22D3,* that promote the growth and survival of the PEL-LCs as tumors (Fig 5B). The role for the peritoneum in molding viral and cellular gene expression of PEL-LCs and PEL cell lines therefore likely contributes to the characteristic development of PELs in body cavities.

PEL-LCs require both KSHV and EBV to be transformed and represent the only available model for PELs co-infected with KSHV and EBV. Recent studies have found that ~70% of 70 cases of classic PELs are co-infected [45–47]. Our characterization of PEL-LCs therefore provides insights into the likely development of PELs. We envision, during the early stages of development of PELs, a transition from PEL-LCs in which paracrine signaling supports their growth to autocrine signaling at their later stages. This transition would be accompanied by changes in EBV gene expression (Fig 4A and S2 Table) from type III which fosters STAT signaling to type I in which KSHV genes make larger relative contributions to survival and growth supported by the increased number of KSHV genomes per cell. This altered expression of viral genes is also mediated by its being favored in the microenvironment of the peritoneum underscoring a pivotal role for body cavities in the development of this lymphoma. PEL-LCs share many features with PEL biopsies and cell lines derived from them. Their examination *in vitro* and *in vivo* has and will identify mechanisms likely to underly the pathogenesis of PEL.

## Methods

### Ethics statement

Protocols for animal work were approved by the UW-Madison SMPH Institutional Care and Use Committee (Protocol M005871). Study protocols for the acquisition and use of primary human samples were reviewed and deemed exempt by the UW-Madison Minimal Risk Institutional Review Board.

### Human peripheral and tonsillar B cells

Peripheral blood B lymphocytes were isolated from buffy coats obtained from Interstate Blood Bank (Memphis, TN). Tonsillar B lymphocytes were purified from remnant surgical tissue obtained from the Cooperative Human Tissue Network (CHTN). Single cell suspensions were isolated from tonsils by maceration of tissue in 1X DPBS+EDTA and passage through a cell strainer. PBMCs were isolated from buffy coats and lymphoid mononuclear cells from tonsillar suspensions through density gradient separation with Ficoll-Paque (GE Healthcare Life Sciences, Piscataway NJ). B lymphocytes were subsequently purified from single-cell suspensions by negative selection with the B cell Isolation Kit II and AutoMACS (Miltenyi Biotech, Auburn CA) following manufacturer’s instructions.

### Animals

Immunodeficient NOD-scid IL2Rg^null^ (NSG) mice (6–12 week old, male or female) used in this study were provided by the UW-Madison Biomedical Research Model Services. Mice were housed in accordance with guidelines approved by the Association for Assessment of Laboratory Animal Care and the University of Wisconsin-Madison School of Medicine and Public Health.

### Cell culture

Co-transformed cells from donors 1 and 2 described previously [9] were subsequently passaged to derive some of the immortalized lines used in this study while others were newly generated. PEL lines (BC-1 and BCBL-1), MC116 (a gift from Shigeki Miyamoto, UW-Madison) and co-transformed cells were cultured in RPMI-1640 (Gibco) supplemented with 10% FBS (Hyclone) and antibiotics (200 U/mL penicillin and 200 ug/mL streptomycin) and maintained at 37oC in a 5% CO2 humidified incubator. The identities of PEL cell lines were confirmed by STR analysis performed by the UWCCC TRIP Lab.

### Lentiviral production and transduction

Lentivirus was generated as previously described [48] using transfection of HEK-293T producer cells.

### KSHV and EBV virus stocks preparation

KSHV and EBV virus stocks were prepared as previously described [9]. KSHV was generated from iSLK-BAC16 cells (kindly provided by Jae U. Jung, Cleveland Clinic, Cleveland, Ohio). Clones of iSLKBAC16 cells were isolated and were induced with 1 μg/mL Doxycycline and 1 mM valproic acid in the absence of G418, Puromycin, and Hygromycin. The supernatants were harvested at days 4 and 8 and cleared of cells and debris by multiple rounds of centrifugation (500 × g for 10 min followed by 3,000 × g for 10 min). EBV was isolated as follows: B95-8 cells were grown in RPMI supplemented with 10% FBS and were induced when they reached 7 × 105 cells/mL with 20 ng/mL TPA and 3.5 mM sodium butyrate. The supernatant was collected at day 5 and cells and debris were removed by centrifugation at 500 × g for 10 min and filtration with a 0.8-μm low-protein binding filter. All viruses were maintained at a pH of 7.2 by addition of HEPES when necessary. KSHV and EBV viral supernatants were concentrated 100-fold by centrifugation at 48,000 × g for 2 h and viral pellets were resuspended at 4°C overnight in RPMI supplemented with 10% FBS and 50 mM HEPES. Separate aliquots were kept at −80 °C and thawed immediately before use; another round of centrifugation at 950 × g for 10 min was performed before use for KSHV viral preparations to remove any remaining debris.

### Infection of B cells with EBV and KSHV

Up to 10^7^ purified B cells were used per condition. In all experiments, a fraction of the cells was left uninfected as a negative control and no cell growth or GFP expression were observed in the absence of infection. During incubation, 50 mM HEPES was added to the culture medium of the B cells. For exposure to KSHV, the wild-type BAC16 virus was added to B cells at an MOI of 2–4 (as measured on 293 cells) and cells and virus were centrifuged at 950 × g for 90 min at room temperature in a volume of 1–2 ml in 12 or 24 well plates. Following spinoculation, cells were washed to remove unbound viruses and resuspended in their culture medium. For co-infection, EBV was added immediately to the KSHV-infected B cells at 1–2 x10^6^ per ml at an MOI of 2–4 (such that more than 90% of exposed B cells are infected with EBV), incubated at 37°C overnight, and then diluted to 5 x10^5^ per ml and observed over time.

### Flow cytometry

The fraction of tonsillar B cells infected with KSHV-BAC16-GFP was determined by flow cytometry. At the indicated timepoints after exposure to virus (described previously), cells were harvested and stained with DAPI (Invitrogen) as a viability dye. For each condition, a minimum of 4,000 cells were acquired on a LSR Fortessa (BD Biosciences, Franklin Lakes NJ) or BD FACSAria II at the UWCCC Flow Cytometry Lab. Data analysis was performed using FlowJo (BD).

For florescence sorting of cells, cells were resuspended in RPMI-1640 (Gibco) supplemented with 10% FBS (Hyclone), antibiotics, 25mM HEPES (Gibco) and DAPI (Invitrogen) as a viability dye and sorted with a BD FACSAria II. For cloning, EGFP-positive cells were sorted into 96-well plates seeded with fibroblast feeder cells and outgrowing EGFP-positive wells expanded to isolate clones.

Immunoglobulin light-chain expression was assayed by first staining for viability with Ghost Dye 780 (Cytek Biosciences, Fremont CA) per manufacturer’s instructions followed by fixation of cells in 4% formaldehyde for 15 minutes at room temperature, washing in excess 1X DPBS, incubation in 0.3% Triton X-100 at room temperature for 10 minutes, washing in 1X DPBS, and resuspending in 100uL 1X DPBS. Following blocking with Human TruStain FcX (Fc Receptor Blocking Solution, Biolegend 422301) samples were stained with PE anti-human Ig light chain κ Antibody (Biolegend 316507) and APC anti-human Ig light chain λ Antibody (Biolegend 316609). Stained samples were acquired on a BD LSR Fortessa. FlowJo was used for analysis of flow cytometry data.

### CRISPR/RfxCas13d targeting

RfxCas13d from pXR001 (Addgene plasmid 109049) was amplified by PCR and cloned into a derivative of lentiCRISPRv2 blast (Addgene plasmid 98293) by Gibson Assembly to generate RfxCas13d-Blast (p4607). Lentivirus was generated as described previously and used to transduce BJAB or immortalized co-transformed cells. A library of RfxCas13d guides (S3 Table) was designed from two sources: nontargeting and GFP-targeted guides from Wessels, et. al. [49] and guides targeting viral and cellular targets identified with the cas13design tool (https://cas13design.nygenome.org/). An oPool (IDT) containing the identified guides was then amplified by PCR and cloned into p4605 (a derivative of lentiCRISPRv2, Addgene plasmid 52961) where the Cas9 ORF has been replaced with mScarlet and the guideRNA expression cassette replaced with a RfxCas13d guideRNA expression cassette) and lentivirus was generated as described. Cells were transduced at an MOI of approximately 0.3 and two harvests were collected (48 hours after transduction and approximately 10 doublings later). Cells were harvested and genomic DNA isolated using the Quick DNA Kit (Zymo, Irvine CA). Two-step PCR amplification with NEBNext UltraII Master Mix (New England Biolabs, Ipswich MA) was used to prepare libraries. Prepared libraries were sequenced on a NovaSeq X Plus by the University of Wisconsin Biotechnology Center DNA Sequencing Facility. Raw reads were processed with cutadapt v4.5 to remove flanking and adapter sequences followed by read count summarization and abundance analysis using the RRA method of Mageck v0.5.9.2.

### Induced immortalization with hTert

A retroviral vector encoding hTert was constructed by cloning the hTert ORF into p3313 (a MMLV retrovector: MCS-IRES-mRFP). Prepared retrovirus was used to transduce co-infected cells. Transduced RFP^+^ cells were sorted as described above and subsequently maintained in complete medium.

### Conditioned medium assay

Minimal medium, consisting of RPMI-1640 (Gibco), 0.25mg/mL bovine serum albumin (VWR), 2.5ug/mL transferrin (Sigma), and 5ug/mL gentamicin (Life Technologies) was used to make conditioned media [14]. The conditioned media were made by incubating either of two clones of co-transformed cells at 2 X 106 cells/mL in minimal medium for 72 hours. Following incubation, the cells were removed by centrifugation and filtration and the conditioned medium diluted 1:1 with minimal medium. The growth of the co-transformed cells seeded at 5 X 104/mL was assayed in at least two experiments with CellTiter-Glo (Promega, Fitchburg WI) both with minimal media and with two stocks of conditioned media.

### Multiplex cytokine assay

Conditioned medium was frozen at -80^o^C and multiplexed cytokine analysis performed on a Luminex 200 system (Luminex, Austin, TX, USA) by Eve Technologies Corp. (Calgary, Alberta) using MILLIPLEX panels (MilliporeSigma, Burlington MA). Subsequent analyses were performed on log2 transformed MFI, Median Fluorescent Intensity, provided in S2 Table. Comparisons between groups was performed using Two-tailed Welch’s t-tests followed by FDR correction for multiple testing with the Benjamini-Hochberg procedure (SciPy Stats v1.9.0) using the number of analytes detected above the LOD (limit of detection; defined as the mean of unconditioned medium + 3 SD) in at least one sample.

### Small molecule inhibitors

Cells were grown in complete medium (RPMI-1640 supplemented with FBS and antibiotics as described previously) or complete medium supplemented with 100nM Rapamycin (Tocris Bioscience, Minneapolis MN) or 100nM Pacritinib (MedChem Express, Monmouth Junction NJ) for 48 hours. Cell number was assayed at 0 and 48 hours with CellTiter-Glo and growth with small molecule treatment calculated relative to complete medium.

### Fluorescence in-situ hybridization (FISH)

Samples were fixed and hybridized as previously described [12]. Viral genomes were scored on an Axiovert 200M microscope and images acquired on an Axio Imager M2 with AxioVision (Zeiss, White Plains, NY).

### Peritoneal xenografting

PEL or co-transformed cells from cell culture were resuspended in 1X sterile PBS (5x10^5^ or 5x10^6^ cells in 100uL) and injected intraperitoneally. Weight was monitored weekly until mice were euthanized for peritoneal lavage collection (18–27 days after injection for single-passage). Xenograft cells were harvested by flushing peritoneal cavities with 3mL 1X sterile PBS. Samples were filtered through a cell strainer, diluted in RBC Lysis Buffer, pelleted and resuspended in 0.5% BSA/DPBS. Host (mouse) cells were removed with a Mouse Xenograft depletion kit (Miltenyi Biotech). Xenografts were classified as tumors when, after accounting for loss due to processing, more cells were recovered than were injected.

### Library preparation and high-throughput sequencing

Samples intended for RNA analysis were harvested into TRIzol (Thermo Fisher) and total RNA isolated with a Direct-zol RNA Microprep kit (Zymo). Library preparation and sequencing were performed by the OMRF Clinical Genomics Center (Oklahoma City, OK) or Yale Center for Genome Analysis (New Haven, CT). mRNA libraries were generated with pA-selection followed by sequencing on an Illumina NovaSeq 6000 (PE101 or PE150). Small RNA libraries were prepared by OMRF with a QIAseq miRNA library prep kit and sequenced on an Illumina NextSeq 500 (SR75). Sequencing data has been deposited to SRA (BioProject PRJNA1007756).

### mRNA-Seq analysis

mRNA-Seq analysis was performed as previously described [9,50] with modifications. Briefly, FASTQ files were aligned to the human (GDC GRCh38) and mouse (mm10) genomes using STAR 2.7.6a. Disambiguate v1.0 was then used to identify human reads. FeatureCounts v2.0.1 was used to quantify host (human) gene expression while viral gene expression was calculated using the UCDS method [9]. Transcripts per million for host genes were determined using TPMCalculator [51] with the disambiguated bam files.

### Small RNA-Seq analysis

Small RNA-Seq analysis was performed using miRge3.0 [52] with viral miRNAs [53–55] from miRbase [56,57].

### RT-PCR for EBV latency transcripts

Samples were harvested into TRIzol (Thermo Fisher) and total RNA isolated with a Direct-zol RNA Microprep kit (Zymo). First strand synthesis with 50ng of total RNA was performed using a ProtoScript II First Strand cDNA synthesis kit (NEB) with a gene-specific primer [58] (5’-CATTTCCAGGTCCTGTACCT-3’). 1 microliter of RT product was used as input for 35 cycles of PCR using Q5 Polymerase (NEB) with a common reverse primer in the K exon (5’-CCCCTCGTCAGACATGAT-3’). Forward primers bound in either the Q exon (5’-AAGGCGCGGGATAGCGT-3’) or the Y3 exon [59] (5-TGGCGTGTGACGTGGTGTAA-3’). PCR products were subsequently separated by agarose gel electrophoresis, imaged on an Azure Imager (Azure Biosystems), and quantified in Fiji (ImageJ 1.51w).

### Statistics

Statistical analyses not otherwise described were performed with Mstat v7.0.1 (Norman Drinkwater, UW-Madison).

## Supporting information

S1 Fig Flow cytometry to detect immunoglobulin λ and Κ light chains. Analysis of mixed populations of co-infected cells and cells infected only with EBV, of co-transformed clones, or the λ positive control lymphoma line MC116 are shown.
(TIF)

S2 Fig The number of EBV and KSHV genomes were measured by FISH in a clone of cells infected only with EBV (721), in clones of co-transformed cells from two tonsil donors, in a clone of co-transformed cells immortalized with hTERT, in a clone of co-transformed cells spontaneously immortalized, and in the JSC-1 PEL cell line by FISH. (TIF)

S3 Fig The peritoneum fosters gene expression of PEL cell lines better mimicking that of PEL biopsies as found for co-transformed cells (Fig 3A). The differential gene expression of PEL cell lines engrafted in immunodeficient mice was measured by RNA-seq and compared to that of the PEL cells grown *in vitro*. The measured differential expression was compared to that identified by Klein et al. [19] to characterize that found in biopsies of PELs when compared to gene expression in other B cell lymphomas. Gene Set Enrichment Analysis (GSEA) against the PEL biopsy expression of Klein et al. indicated that changes in gene expression in the PEL cell lines (BC-1 and BCBL-1 combined) grown as tumors relative to that of these cells grown *in vitro* is concordant with the increased genes enriched in PEL biopsies (NES = 2.06, FDRq < 0.001).
(TIF)

S4 Fig (A) Three distinct transcripts encode EBNA-1, driven by three promoters: Cp, Wp, Qp. The most restricted form of latency (latency I) is associated with transcripts driven by Qp. (B) RNA-seq read depth across the locus. (C). RT-PCR was performed using primers shown in (A) and the product intensity normalized to the Y3 product from LCL.
(TIF)

S5 Fig Gene Set Enrichment Analysis performed as in Fig 5 using RNA-Seq data from a second passage of a co-transformed cell clone in the peritoneal cavity.
(TIF)

S1 Table Measurements of viral RNAs by RNA-seq in non-immortalized and immortalized co-transformed cells grown *in vitro* and as tumors in the peritoneal cavities of NSG mice and of PEL cell lines grown *in vitro* and as tumors in the peritoneal cavities of NSG mice. Values represent the mean log2-transformed EBV and KSHV gene expression within indicated subsets of *in vitro* or *in vivo* samples.
(XLS)

S2 Table Measurements using a Luminex assay of cytokines released by cells infected only with EBV, by co-transformed cells, and by PEL cell lines. Values represent log2-transformed Luminex Fluorescence Intensity measurements.
(XLS)

S3 Table CRISPR/RfxCas13d guide RNAs used in this study. (XLS)
