# Supplementary figures and images for: Kaposi sarcoma-associated herpesvirus cooperates with Epstein-Barr virus to co-transform a small set of human B cells oncogenically
Source: PLoS Pathog. 2025 Jun 23;21(6):e1013281. doi: 10.1371/journal.ppat.1013281 (PMC12204619; doi:10.1371/journal.ppat.1013281)

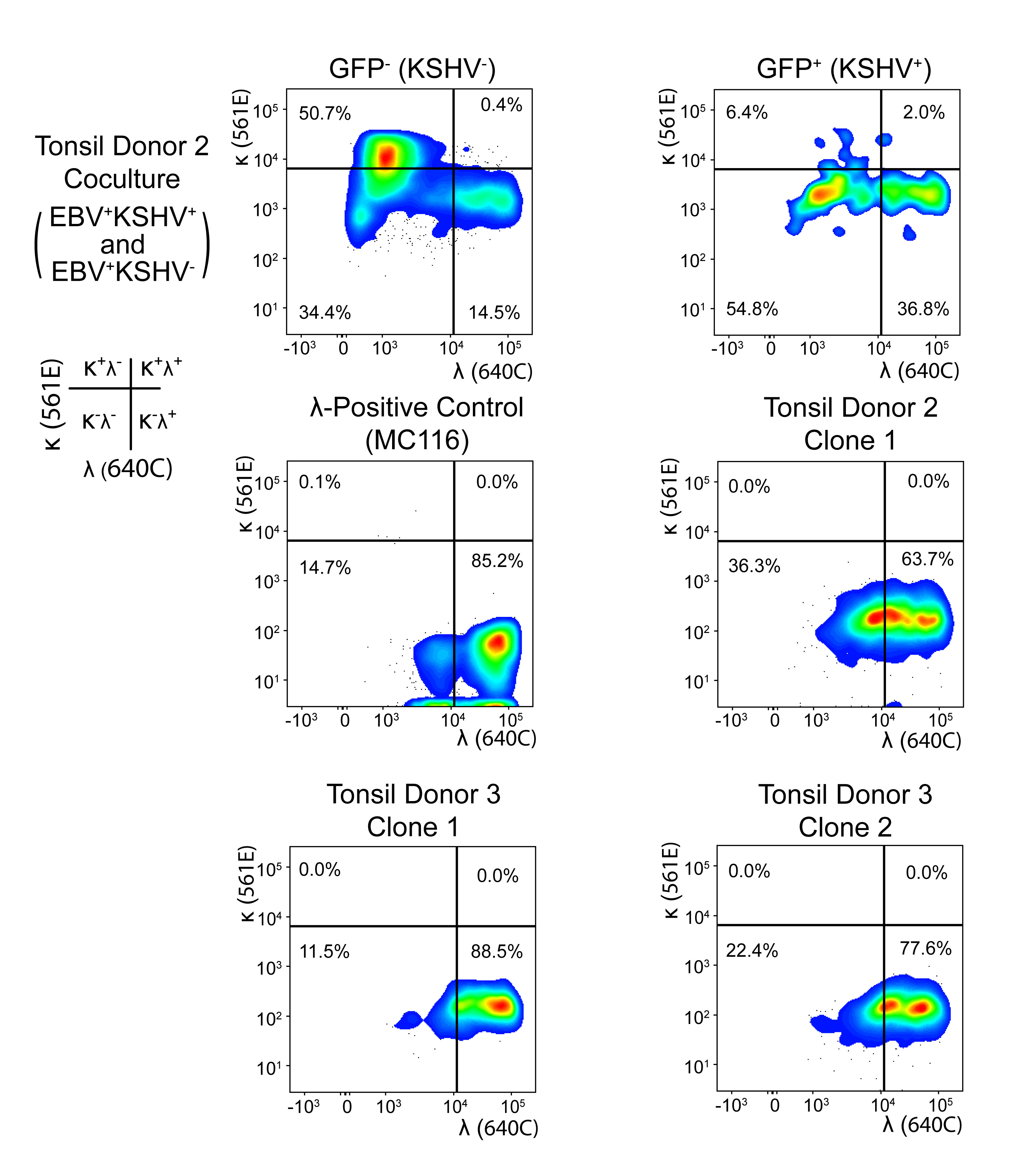

Supplement: S1 Fig — Flow cytometry to detect immunoglobulin λ and Κ light chains. Analysis of mixed populations of co-infected cells and cells infected only with EBV, of co-transformed clones, or the λ positive control lymphoma line MC116 are shown. (TIF) [file ppat.1013281.s001.tif]

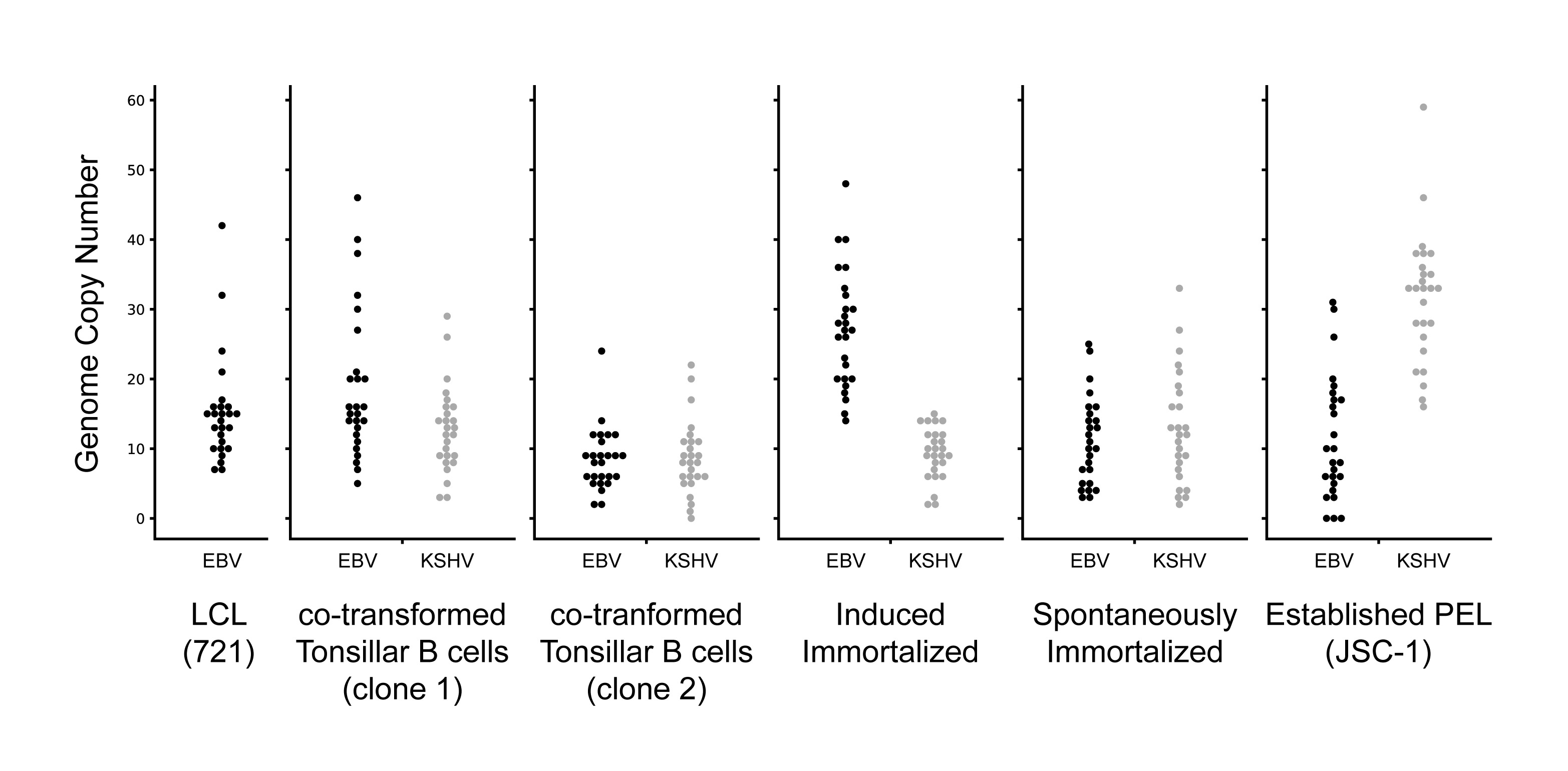

Supplement: S2 Fig — (TIF) [file ppat.1013281.s002.tif]

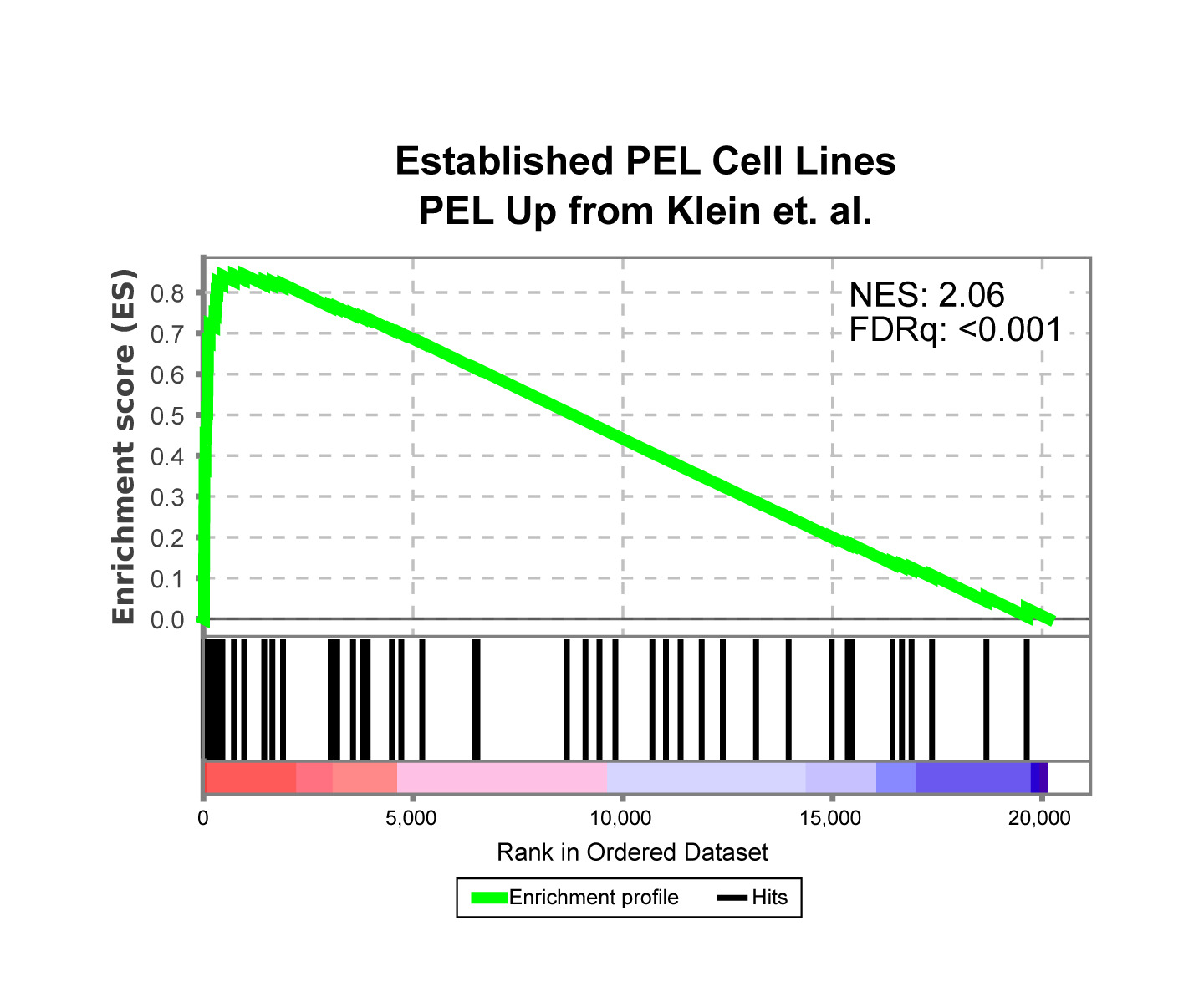

Supplement: S3 Fig — The peritoneum fosters gene expression of PEL cell lines better mimicking that of PEL biopsies as found for co-transformed cells (Fig 3A). The differential gene expression of PEL cell lines engrafted in immunodeficient mice was measured by RNA-seq and compared to that of the PEL cells grown in vitro. The measured differential expression was compared to that identified by Klein et al. [19] to characterize that found in biopsies of PELs when compared to gene expression in other B cell lymphomas. Gene Set Enrichment Analysis (GSEA) against the PEL biopsy expression of Klein et al. indicated that changes in gene expression in the PEL cell lines (BC-1 and BCBL-1 combined) grown as tumors relative to that of these cells grown in vitro is concordant with the increased genes enriched in PEL biopsies (NES = 2.06, FDRq < 0.001). (TIF) [file ppat.1013281.s003.tif]

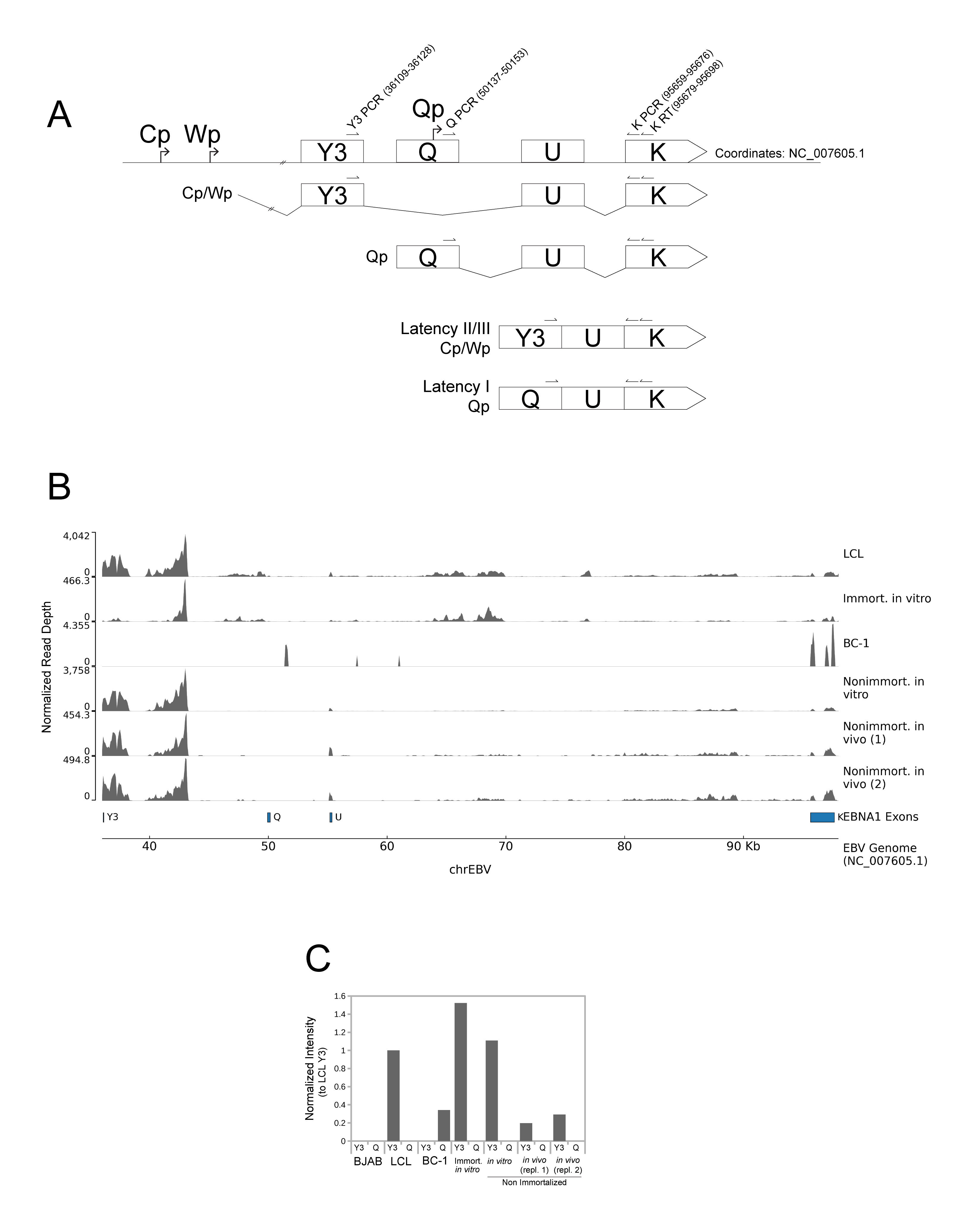

Supplement: S4 Fig — (A) Three distinct transcripts encode EBNA-1, driven by three promoters: Cp, Wp, Qp. The most restricted form of latency (latency I) is associated with transcripts driven by Qp. (B) RNA-seq read depth across the locus. (C). RT-PCR was performed using primers shown in (A) and the product intensity normalized to the Y3 product from LCL. (TIF) [file ppat.1013281.s004.tif]

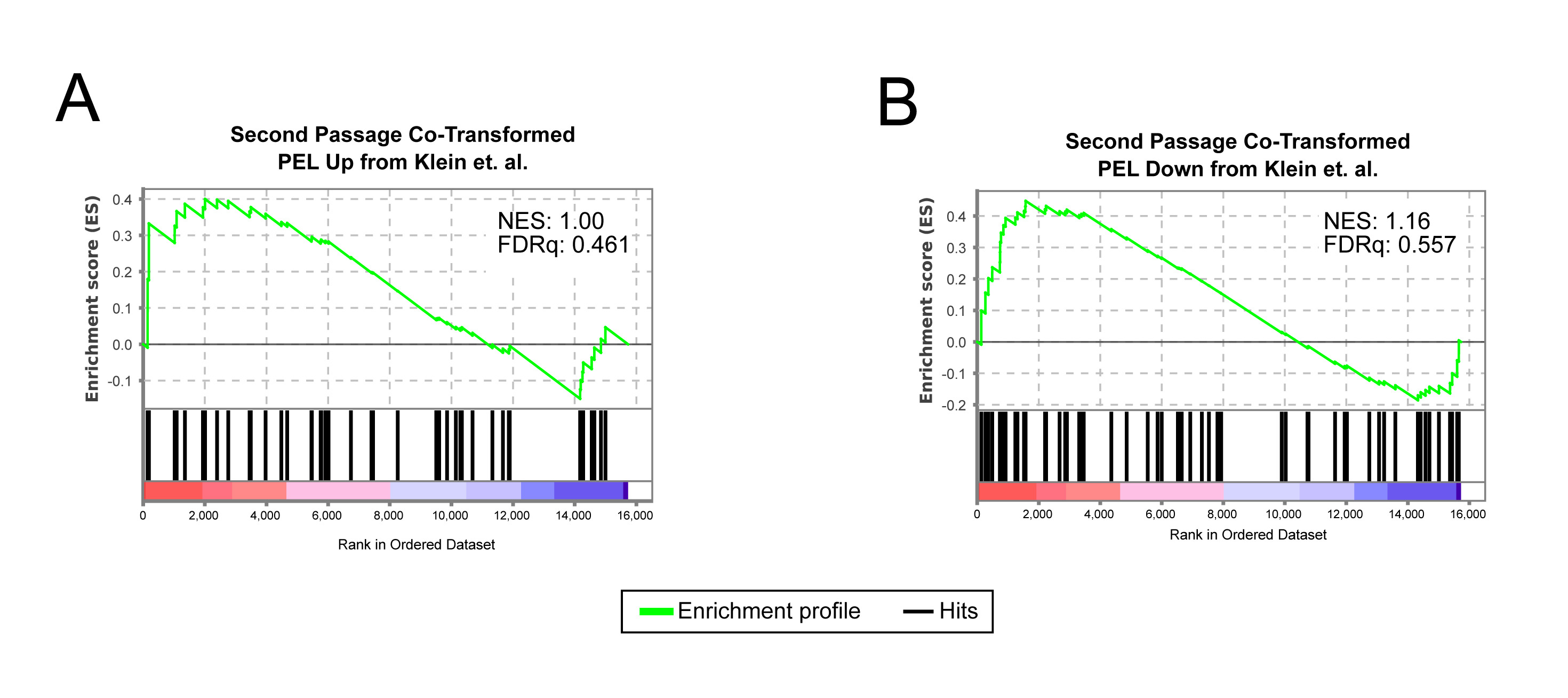

Supplement: S5 Fig — Gene Set Enrichment Analysis performed as in Fig 5 using RNA-Seq data from a second passage of a co-transformed cell clone in the peritoneal cavity. (TIF) [file ppat.1013281.s005.tif]
